# Pre-treatment peripheral blood immunophenotyping and response to neoadjuvant chemotherapy in operable breast cancer

**DOI:** 10.1186/s13058-024-01848-z

**Published:** 2024-06-10

**Authors:** Roberto A. Leon-Ferre, Kaitlyn R. Whitaker, Vera J. Suman, Tanya Hoskin, Karthik V. Giridhar, Raymond M. Moore, Ahmad Al-Jarrad, Sarah A. McLaughlin, Donald W. Northfelt, Katie N. Hunt, Amy Lynn Conners, Ann Moyer, Jodi M. Carter, Krishna Kalari, Richard Weinshilboum, Liewei Wang, James N. Ingle, Keith L. Knutson, Stephen M. Ansell, Judy C. Boughey, Matthew P. Goetz, Jose C. Villasboas

**Affiliations:** 1 Department of Oncology, Mayo Clinic, Rochester, MN USA; 2 Division of Hematology, Mayo Clinic, Rochester, MN USA; 3 Department of Quantitative Health Sciences, Mayo Clinic, Rochester, MN USA; 4 Department of Surgery, Mayo Clinic, Jacksonville, FL USA; 5 Division of Hematology and Oncology, Mayo Clinic, Scottsdale, AZ USA; 6 Department of Radiology, Mayo Clinic, Rochester, MN USA; 7 Department of Laboratory Medicine and Pathology, Mayo Clinic, Rochester, MN USA; 8 Department of Laboratory Medicine and Pathology, University of Alberta, Edmonton, Alberta Canada; 9 Schulze Center for Novel Therapeutics, Mayo Clinic, Rochester, MN USA; 10 Department of Immunology, Mayo Clinic, Jacksonville, FL USA; 11 Department of Surgery, Mayo Clinic, Rochester, MN USA

**Keywords:** Breast cancer, Immunology, Biomarkers, Chemotherapy, Translational research, Single cell technologies

## Abstract

**Background:**

Tumor immune infiltration and peripheral blood immune signatures have prognostic and predictive value in breast cancer. Whether distinct peripheral blood immune phenotypes are associated with response to neoadjuvant chemotherapy (NAC) remains understudied.

**Methods:**

Peripheral blood mononuclear cells from 126 breast cancer patients enrolled in a prospective clinical trial (NCT02022202) were analyzed using Cytometry by time-of-flight with a panel of 29 immune cell surface protein markers. Kruskal–Wallis tests or Wilcoxon rank-sum tests were used to evaluate differences in immune cell subpopulations according to breast cancer subtype and response to NAC.

**Results:**

There were 122 evaluable samples: 47 (38.5%) from patients with hormone receptor-positive, 39 (32%) triple-negative (TNBC), and 36 (29.5%) HER2-positive breast cancer. The relative abundances of pre-treatment peripheral blood T, B, myeloid, NK, and unclassified cells did not differ according to breast cancer subtype. In TNBC, higher pre-treatment myeloid cells were associated with lower pathologic complete response (pCR) rates. In hormone receptor-positive breast cancer, lower pre-treatment CD8 + naïve and CD4 + effector memory cells re-expressing CD45RA (T_EMRA_) T cells were associated with more extensive residual disease after NAC. In HER2 + breast cancer, the peripheral blood immune phenotype did not differ according to NAC response.

**Conclusions:**

Pre-treatment peripheral blood immune cell populations (myeloid in TNBC; CD8 + naïve T cells and CD4 + T_EMRA_ cells in luminal breast cancer) were associated with response to NAC in early-stage TNBC and hormone receptor-positive breast cancers, but not in HER2 + breast cancer.

**Trial registration:**

NCT02022202. Registered 20 December 2013.

**Supplementary Information:**

The online version contains supplementary material available at 10.1186/s13058-024-01848-z.

## Introduction

The successful implementation of immunotherapy in multiple cancers has led to an increased appreciation of the relevance of antitumor immune responses in clinical outcomes. In patients with breast cancer, the generation of anticancer adaptive immunity appears more robust in the triple-negative (TNBC) and the human epidermal growth factor receptor 2 (HER2)-positive subtypes, while estrogen receptor (ER)-positive/HER2-negative breast cancers (herein referred to as luminal subtype) are generally regarded as less immunogenic [1, 2]. The robustness of immune cell infiltration within the tumor stroma is both prognostic and predictive of response to chemotherapy and immunotherapy in all breast cancer subtypes [3–5]. Furthermore, robust tumor immune cell infiltration is highly associated with favorable prognosis in patients with early-stage TNBC, even without systemic therapy administration [6, 7].

Most of our understanding of the interactions between breast cancer tumor cells and immune cells comes from “tumor-centric” research evaluating immune cells infiltrating the tumor microenvironment. However, immune cells infiltrating tumors must first be recruited from the peripheral blood systemic pool. Akin to the use of “liquid biopsies” to detect circulating tumor DNA, studies in other malignancies [8, 9] and in breast cancer [10, 11] have demonstrated that distinct peripheral blood immune signatures at the time of diagnosis (before any treatment) and changes in those signatures induced by treatment have the potential to predict treatment outcome.

**Fig. 1:**
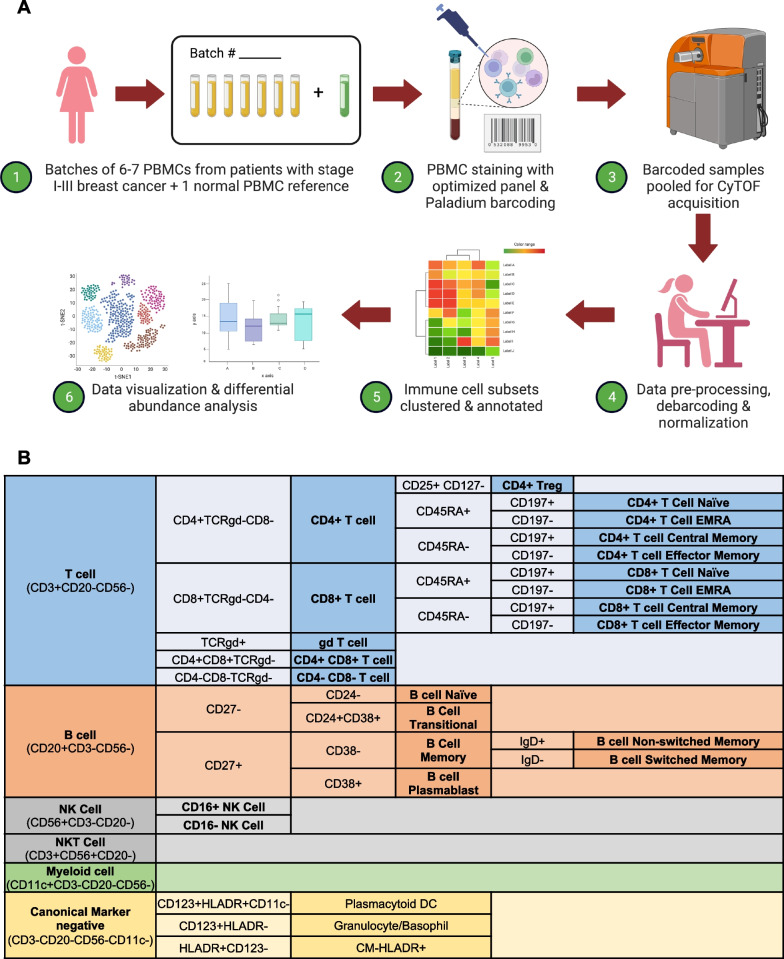
(**A**) Peripheral blood mononuclear cell immune phenotyping workflow, (**B**) Labeling strategy

Comprehensive simultaneous enumeration of distinct peripheral blood immune cell subpopulations has been historically limited by the low-plex capabilities of technologies such as standard flow cytometry. However, the advent of highly multiplexed proteomic platforms, such as mass cytometry (also known as Cytometry by Time-Of-Flight [CyTOF]), has enabled the simultaneous investigation of large numbers of cell markers at single-cell resolution. By replacing fluorophores with non-organic elements, mass cytometry offers an extensive spectrum with minimal spillover between channels and virtually no biological signal background [12], positioning CyTOF as an ideal technology to characterize the systemic immunological landscape of patients with cancer [12]. In this study, we aimed to evaluate the relative abundance of the major peripheral blood immune cell lineages (i.e., B, T, NK, and myeloid cells)—and their diverse subsets—in patients with operable breast cancer treated with neoadjuvant chemotherapy (NAC) within the context of a prospective clinical trial [13]. To accomplish this, we used a CyTOF panel including 29 surface protein markers (Fig. 1) to interrogate the profile of peripheral blood mononuclear cell (PBMC) samples obtained before initiation of neoadjuvant chemotherapy (NAC) and evaluate the differential abundance of immune cell subsets according to breast cancer subtype and pathologic response.

## Materials and methods

### Patient population

PBMC samples were prospectively collected from 126 of 132 eligible patients enrolled in the Breast Cancer Genome-Guided Therapy study at Mayo Clinic (NCT02022202) between March 5, 2012, and May 1, 2014. Patients with a new diagnosis of operable invasive breast cancer of any subtype were eligible if the primary tumor measured ≥ 1.5 cm, and they were recommended to receive NAC by their treating oncologist. The primary results of the study, including patient characteristics and genomic profiling data, have been published previously [13]. Clinical approximated breast cancer subtypes were defined using the St. Gallen Criteria [14]: luminal A (ER > 10% + grade 1 or ER > 10% + grade 2 + Ki-67 < 15%); luminal B (ER > 10% + grade 2 + Ki67 ≥ 15% or ER > 10% + grade 3); HER2 + (defined as 3 + by immunohistochemistry [IHC] or amplified by fluorescence in situ hybridization [FISH]); and TNBC (ER ≤ 10%, progesterone receptor ≤ 10%, and HER2-).

Participants in this study were recommended to receive twelve doses of weekly paclitaxel (with trastuzumab for HER2 + breast cancer), followed by four cycles of an anthracycline-based regimen. Pertuzumab was allowed along with trastuzumab for HER2 + breast cancer after September 2012. Carboplatin was allowed for TNBC after June 2013. None of the patients enrolled in this study received immunotherapy. Following completion of NAC, patients underwent surgery, and resected tissue was evaluated for pathologic response. Pathologic complete response (pCR) was defined as the absence of invasive tumor in the breast and axillary lymph nodes (ypT0/Tis, ypN0). The amount of residual disease after NAC was evaluated using the Residual Cancer Burden (RCB) index, with RCB-0 representing pCR, and RCB-1, RCB-2, and RCB-3 representing increasing amounts of residual disease [15, 16]. Endocrine therapy was to be administered postoperatively for patients with ER + breast cancer. The Mayo Clinic Institutional Review Board and appropriate committees approved this study. All patients provided written informed consent.

### PBMC collection and storage

PBMC suspensions were prospectively created from peripheral blood collected using heparin tubes (Becton Dickinson Vacutainer® SKU: 367874) before NAC initiation by the Mayo Clinic Biospecimens Accessioning and Processing laboratory. Mononuclear cells were isolated using a density gradient isolation technique. Following isolation, the sample was viably cryopreserved in a mixture of cell culture medium, fetal bovine serum (FBS), and dimethyl sulfoxide (DMSO). Cells were subsequently slow frozen to maintain cell integrity and stored in liquid nitrogen.

### Mass cytometry staining

We divided the study population into three cohorts according to breast cancer subtype: TNBC, HER2-positive, and luminal. For each cohort, samples were thawed and processed in batches of 6–7 individual patient samples, along with a longitudinal reference sample, using the workflow depicted in Fig. 1A. The longitudinal reference samples were technical replicates created from a single PMBC pool composed of four healthy donors. These reference samples were used for panel titration and served as a longitudinal reference to identify issues with antibody staining quality and batch effects [17, 18]. The order in which each patient sample was processed within each cohort was determined by randomization, stratified by pCR status.

After thawing, samples were stained with a panel of 29 commercially available, metal-tagged antibodies (Fluidigm, CA) optimized to identify major human immune cell subsets (Fig. 1B). Final antibody concentrations were selected based on signal-to-noise ratio and their ability to differentiate negative, dim, and bright populations. Samples were stained individually using standard manufacturer protocol (Fluidigm, CA), barcoded overnight with a unique palladium barcode during DNA intercalation, and pooled for acquisition in the mass cytometer.

### Identification of individual immune cell populations

After acquisition in the mass cytometer, output data was de-barcoded and normalized on a per-batch basis to the median intensity of Eqbeads [19]. Gaussian discrimination parameters were used for data cleanup [20]. Flow Cytometry Standard (FCS) files were uploaded to an automated platform for unbiased processing (Astrolabe Diagnostics, Arlington, VA, USA), which uses the flow self-organization map (FlowSOM) algorithm [21] followed by a labeling step to automatically assign cells to pre-selected and biologically known immune cell lineages. Patient-level metadata was added to the experimental matrix, and immune cell subsets were clustered and annotated to determine the differential abundance of immune cell subpopulations across clinical and pathological groups of interest.

First, we identified and calculated the frequencies of major immune cell populations (i.e., B, T, NK, and myeloid cells) according to lineage-defining cell surface proteins (Fig. 1B). Within these major immune cell compartments, we evaluated the individual cell maturation and antigen-experienced states of T and B cells and distinct NK cell subsets according to the labeling strategy shown in Fig. 1B. Of note, CD11c was used to define the myeloid lineage in these experiments, due to suboptimal performance of CD14 and CD16 (which were thus excluded from the labeling hierarchy). Due to this, no additional phenotyping of this compartment was carried out. Percent of immune cell subsets is presented here as a percent of all PBMCs.

### Data visualization and statistical analyses

For an initial exploration of the high-dimensional data generated in this study, we utilized the Uniform Manifold Approximation and Projection (UMAP) technique for dimensionality-reduction algorithm [22]. We projected PBMC data from all patients, according to each breast cancer subtype, and according to responses to systemic therapy into UMAP plots generated using OMIQ (Dotmatics, Boston, MA). Kruskal–Wallis tests or Wilcoxon rank-sum tests were used to assess whether an immune cell type (expressed as a percent of the total immune cells) differed with respect to breast cancer subtype. Wilcoxon rank-sum tests were used to compare patients with and without pCR in the HER2 + and TNBC subtypes. Given the expected low rates of pCR after NAC in the luminal breast cancer subtype, we grouped patients with pCR and minimal residual disease after NAC (RCB index class 0/1) versus those with moderate-to-extensive residual disease (RCB class 2/3). *p* values < 0.05 were considered statistically significant. Since the analysis was exploratory, no correction for multiple comparisons was performed. Analysis was performed using SAS (Version 9.4, SAS Institute, Inc. Cary, NC).

## Results

### Experimental efficiency and PBMC phenotyping

Viably cryopreserved PBMC samples from 126 patients obtained before the initiation of NAC were available. After thawing the cryopreserved samples, the average cell count was 3.94 × 10^6^ (SD 1.94 × 10^6^), with mean post-thaw cell viability of 81% (SD 15%). After acquisition on the mass cytometer, the mean yield per sample was 506,099 single-cell events (range: 48,725–1,130,427). Four samples (3 from patients with luminal breast cancer and one from TNBC) were excluded from subsequent analyses due to a low number of single-cell events, leaving 122 evaluable samples. In these, we analyzed a total of 61,744,075 single-cell events (luminal: 28,465,649; TNBC: 13,906,902; and HER2-positive: 19,371,524). The average yield (SD) per sample by breast cancer subtype was luminal: 605,652 (217,935); TNBC: 356,587 (239,863); and HER2-positive: 538,098 (284,120).

### Patient and tumor characteristics

Of the 122 evaluable samples, 47 (38.5%) were from patients with luminal breast cancer (11 luminal A, 36 luminal B, 2 luminal subtype unknown), 39 (32%) from patients with TNBC, and 36 (29.5%) from patients with HER2 + breast cancer (16 ER + /HER2 + and 20 ER-/HER2 +). Baseline patient characteristics from each cohort and their best response to NAC are detailed in Table 1. Patients with TNBC included in this study were more frequently clinically node-negative (cN0) at presentation compared to patients with other breast cancer subtypes (64% cN0 in TNBC compared to 34% and 22% for luminal and HER2 +, respectively). Stromal TILs were available in 24 (62%) of patients with TNBC. The median TIL level was 20% (range 1–80%, IQR 10–40%). TIL levels were not obtained for the luminal or HER2 + breast cancer cohorts (Table 1).

**Table 1:** Patient characteristics

	HER2 (N = 36)	Luminal (N = 47)	TNBC (N = 39)
*Age*			
Median (IQR)	53 (39–63)	48 (41–55)	52 (46–59)
Range	30–73	21–71	32–73
*Menopausal status*			
Pre	17 (47.2%)	28 (59.6%)	14 (35.9%)
Peri	1 (2.8%)	2 (4.3%)	2 (5.1%)
Post	17 (47.2%)	15 (31.9%)	19 (48.7%)
Unknown	1 (2.8%)	2 (4.3%)	4 (10.3%)
*Clinical T stage*			
T1	4 (11.1%)	3 (6.4%)	5 (12.8%)
T2	9 (25.0%)	23 (48.9%)	21 (53.8%)
T3	21 (58.3%)	19 (40.4%)	12 (30.8%)
T4	2 (5.6%)	2 (4.3%)	1 (2.6%)
*Clinical N stage*			
N0	8 (22.2%)	16 (34.0%)	25 (64.1%)
N1	25 (69.4%)	29 (61.7%)	12 (30.8%)
N2	2 (5.6%)	1 (2.1%)	1 (2.6%)
N3	1 (2.8%)	1 (2.1%)	1 (2.6%)
*Clinical node status*			
Negative	8 (22.2%)	16 (34.0%)	25 (64.1%)
Positive	28 (77.8%)	31 (66.0%)	14 (35.9%)
*Grade*			
1–2	15 (41.7%)	34 (72.3%)	7 (17.9%)
3	21 (58.3%)	13 (27.7%)	32 (82.1%)
*Neoadjuvant regimens*^a^			
T → AC	0	47	37
TCb → AC	0	0	2
TH + / − P → AC or EC or FEC	35	0	0
DHP → AC	1	0	0
*pCR*			
Yes	16 (44.4%)	4 (8.5%)	21 (53.8%)
No	20 (55.6%)	43 (91.5%)	18 (46.2%)
*RCB class*			
0/1	21 (58.3%)	7 (14.9%)	27 (69.2%)
2/3	15 (41.7%)	38 (80.9%)	10 (25.6%)
Not obtained	0	2 (4.3%)	2 (5.1%)
*Stromal TILs*			
Not obtained	36	47	15
Median (IQR)			20% (10–40%)
Range			1–80%

^a^T, Paclitaxel; A, Doxorubicin; C, Cyclophosphamide; Cb, Carboplatin; D, Docetaxel; E, Epirubicin; F, 5-Fluorouracil; H, Trastuzumab; P, Pertuzumab

### Pre-treatment peripheral blood immune phenotype according to breast cancer subtype

**Fig. 2:**
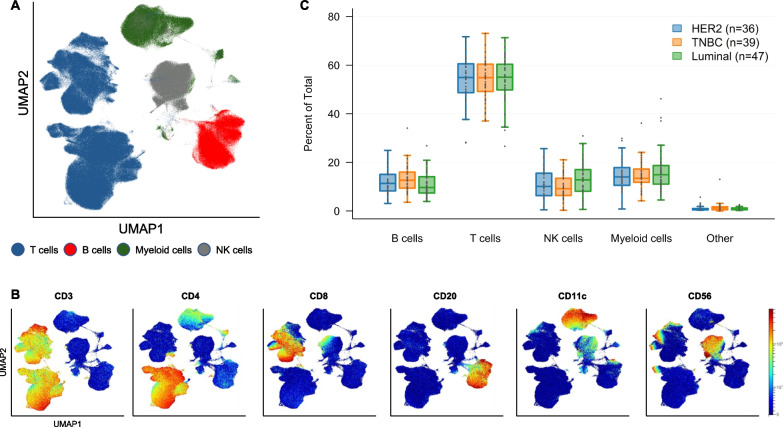
Major immune cell compartments in the overall study population. (**A**) UMAP projection of major PBMC immune cell compartments, (**B**) Canonical marker expression of in each island corresponding to panel (**A**), (**C**) Relative pre-treatment abundance of major immune cell populations according to breast cancer subtype

For visualization purposes, we projected all CD45 + viable single-cell events into a UMAP and identified major immune cell islands according to the expression of lineage-defining markers (Fig. 2A, B). We calculated the total frequencies of the major immune cell subtypes across the three breast cancer subtypes (Fig. 2C). Across breast cancer subtypes, the largest peripheral blood immune cell compartment was the T cell compartment (CD45 + CD3 + CD20-CD11c-CD56-), followed by overall similar frequencies of B cells (CD45 + CD3-CD20 + CD11c-CD56-), myeloid cells (CD45 + CD3-CD20-CD11c + CD56-), and NK cells (CD45 + CD3-CD20-CD11c-CD56 +). The relative abundances of pre-treatment peripheral blood T cells, B cells, myeloid cells, NK cells, and unclassified cells did not significantly differ according to breast cancer subtype. Additionally, we did not identify significant differences in the phenotypic composition of each of the individual compartments of T cells, myeloid cells, B cells, and NK cells (Supplement Figs. S1–S4 show the distribution of B and T cell subsets according to breast cancer subtype). Within unclassified cells, canonical marker negative (CD3-CD11c-CD20-CD56-CD123-) HLADR + cells were highest in TNBC (TNBC: 0.39%, HER2 + BC 0.28%, and luminal: 0.17%, *p* = 0.0228).

We observed a moderate negative correlation between age and the levels of peripheral blood CD8 + naïve T cells across breast cancer subtypes, with the strongest correlation seen in patients with luminal breast cancers (Spearman rank correlation rho − 0.57 in luminal, − 0.51 in HER2 + and − 0.40 in TNBC). Correlations of other immune cells with age are shown in Fig. S8 and Supplementary Table 1.

### Pre-treatment peripheral blood immune phenotype according to response to NAC within each breast cancer subtype

#### TNBC

**Fig. 3:**
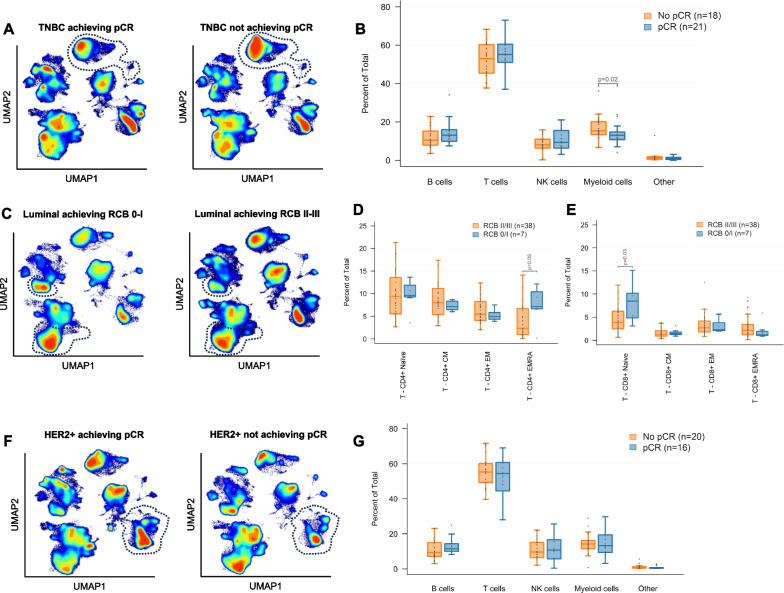
PMBC immunophenotypic differences were observed according to response to neoadjuvant chemotherapy (NAC) in TNBC and luminal breast cancers. (**A**) Density plots showing lower density of myeloid cells (dashed line in patients with TNBC who achieved pCR (left) compared to those who did not (right), (**B**) Relative pre-treatment abundance of major immune cell populations in TNBC according to response to NAC, (**C**) Density plots showing higher density of CD8 + naïve T (dashed outline in top island) and CD4 + TEMRA cells (dashed outline in bottom island) in patients with luminal BC with minimal or no residual disease (RCB 0-I) versus moderate to extensive residual disease (RCB II-III) after NAC, (**D**) Relative pre-treatment abundance of CD4 + T cell subsets in luminal breast cancer according to response to NAC, (**E**) Relative pre-treatment abundance of CD8 + T cell subsets in luminal breast cancer according to response to NAC, (**F**) Density plots showing a trend towards higher density of B cells in patients with HER2 + who achieved pCR (left) compared to those who did not (right), (**G**) Relative pre-treatment abundance of major immune cell populations in HER2 + breast cancer according to response to NAC

Among 39 patients with TNBC, 21 (54%) achieved pCR (Table 1). The distribution of RCB was RCB 0/1: 27 (69%), RCB 2/3: 10 (26%), and not available in 2 (5%). The proportion of pre-NAC myeloid cells (CD3-CD20-CD56-CD11c +) was significantly lower among the patients who achieved a pCR compared to those with residual disease (median 13.1% vs. 15.4%, *p* = 0.0217), Fig. 3A, B. No significant differences in B, T, or NK cells were seen according to response to NAC (Fig. S5). Among the 24 patients with stromal TIL data, TIL levels were not found to differ significantly between patients who achieved pCR (n = 14, median TILs 20%, IQR 10–40%) and those who did not (n = 10, median TILs 25%, IQR 5–30%, *p* = 0.68, Fig. S9). Weak to moderate correlations were observed between stromal TIL levels and specific peripheral blood immune cell populations (Supplementary Table 2 and Figs. S10–S12).

#### Luminal

Among 47 patients with luminal breast cancer (11 luminal A, 36 luminal B), 4 (9%) achieved pCR (Table 1). The distribution of RCB was RCB 0/1: 7 (15%), RCB 2/3: 38 (81%), and not available in 2 (4%) patients (Table 1). All 7 patients who achieved RCB 0/1 had tumors consistent with a luminal B-like phenotype (ER > 10% + grade 3 [2 pts] or ER > 10% + grade 2 + Ki-67 ≥ 15% [5 pts]). No statistically significant differences in the proportion of total myeloid, B, T, or NK cells were detected between patients who achieved pCR versus not, or according to RCB (data not shown). However, within the T cell compartment, CD8 + naïve (CD3 + CD8 + CD45RA + CD197 +) and CD4 + effector memory cells re-expressing CD45RA T cells (T_EMRA_, CD3 + CD4 + CD45RA + CD197-) were significantly higher in patients with better response to NAC (RCB 0/1) compared to those with more extensive residual disease (RCB 2/3, CD8 + naïve median 8.5% vs 3.9%, *p* = 0.0273; CD4 + T_EMRA_ median 7.1% vs 2.4%, *p* = 0.0467, Figs. 3C–E and S6).

#### HER2-positive

Among 36 patients with HER2 + breast cancer, 16 (44%) achieved pCR (Table 1). The distribution of RCB was RCB 0/1: 21 (58%) and RCB 2/3: 15 (42%) (Table 1). Pre-NAC total B cells trended higher among patients who achieved a pCR compared to those with residual disease (median 11.5% vs. 9.3%, *p* = 0.0827), Fig. 3F–G. Within the B cell compartment, transitional B cells were numerically higher among patients who achieved pCR versus not (median 0.89% vs. 0.62%, *p* = 0.0915) (Fig. S7).

## Discussion

It is now well established that antitumor immunity plays a key role in the treatment response and prognosis of patients with breast cancer. The presence of high levels of TILs and of tumor-derived immune-related gene expression are associated with improved prognosis and therapeutic response, particularly in triple-negative and HER2 + breast cancer [1–6, 23]. In addition, morphological immune features identified in regional lymph nodes are also prognostic in TNBC [24, 25]. Based on the hypothesis that tumor-triggered immune responses can be detected not only in the tumor microenvironment and lymph nodes but also in the peripheral blood, this study utilized CyTOF to evaluate the circulating immune cell repertoire of patients with operable breast cancer before initiation of NAC and potential associations with response to NAC. We identified significant differences in the peripheral blood immune phenotype according to treatment response in patients with TNBC and luminal breast cancer (in the myeloid and T cell compartments, respectively). However, among patients with HER2 + breast cancer, pre-NAC B cells only trended higher in patients achieving pCR compared to those with residual disease.

Our findings in the TNBC cohort suggest that higher pre-treatment circulating myeloid cells may be associated with NAC resistance. Myeloid cells, including monocytes, granulocytes, and myeloid-derived suppressor cells (MDSCs) have potent immunosuppressive effects that counteract the endogenous antitumor immune response [26]. Tumor-derived inflammatory signals may promote the expansion of myeloid cells [27], which can, in turn, promote tumor progression by infiltrating tumors or homing to distant organs and establishing pre-metastatic niches that “prime” tissues for the engraftment of disseminating tumor cells [28–30]. It has been shown that myeloid cells are enriched in the tumor microenvironment of chemoimmunotherapy-resistant breast cancer tumors [31, 32]. Additionally, peripheral blood MDSCs are significantly elevated in patients with various cancers compared to unaffected individuals [33], and higher expression of peripheral blood macrophage-related chemokines (e.g. CCL3) have been associated with lower pCR rates in the context of neoadjuvant chemoimmunotherapy [11]. While we were unable to further characterize the myeloid compartment in our study, our data supports further evaluation of circulating myeloid cells throughout NAC in TNBC, particularly considering that tumor-associated myeloid cells exist in a diverse phenotype continuum [34, 35] that may also be reflected in the peripheral blood. Notably, while it has been reported that higher T cell levels within TNBC tumors are associated with pCR after NAC [36], we did not observe statistically significant differences in baseline peripheral blood T cell subsets according to subsequent NAC response. This lack of association may be due to the relatively small TNBC sample size in our study, or due to tumor immune phenotype differences (and their association with treatment response) not being fully recapitulated in the peripheral blood. Additionally, it is possible that peripheral blood T cell dynamics during chemotherapy + / − immunotherapy may be more informative than isolated baseline values (the only available in our study). Indeed, it has been suggested that peripheral blood cytotoxic T cell signatures at the end of NAC may be associated with long-term outcomes among patients with chemotherapy resistant tumors [10].

Patients with luminal breast cancer who achieved a more robust response to NAC exhibited higher levels of pre-NAC naïve CD8 + T cells and of CD4 + T_EMRA_ cells compared to those with more extensive residual disease. These findings are in alignment with previous studies in lung and head and neck cancer, which have demonstrated a positive correlation between higher levels of peripheral blood naïve CD8 + T cells and survival [37, 38]. In young women with luminal breast cancers, higher intratumoral CD8 + T cells correlate with improved survival [39]. Naïve T cells—immune cells that have not yet encountered antigen—can differentiate into several types of effector T cells with the capacity to subsequently destroy cancer cells. Effector CD8 + T cells derived from naïve subsets may be better able to maintain their replicative potential and resist exhaustion compared to CD8 + T cells derived from memory subsets [40]. With regards to CD4 + T_EMRA_ cells, these have been found to be more abundant in the peripheral blood of breast cancer survivors compared to healthy volunteers [41], but associations with response to chemotherapy are less well understood. Further studies confirming these observations in additional cohorts and exploring underlying mechanisms by which these cells contribute to the anti-tumor immune response in luminal breast cancers are needed.

We observed a moderate negative correlation (rho = − 0.57) between age and pre-NAC levels of peripheral blood naïve CD8 + T cells in patients with luminal breast cancer, raising questions on age as a potential confounder in the association of these cells with treatment response. In this cohort, we found that age did not differ significantly between patients achieving RCB 0/1 and those achieving RCB 2/3. However, a larger dataset would be needed to examine the association of age and baseline peripheral blood naïve CD8 + T cells with chemoresistance in patients with luminal breast cancer.

A growing body of literature suggests that B cell immunity is highly relevant in breast cancer, particularly in the HER2 + subtype and in the context of treatment with trastuzumab [42–44]. Higher tumor-infiltrating B cells correlate with improved prognosis in various solid tumors, including melanoma, gastrointestinal tumors, non-small cell lung cancer, and ovarian cancer [45–50]. When compared to healthy controls, patients with breast cancer have higher total peripheral blood B cells, particularly memory B cells [51]. While we did not observe statistically significant differences in total peripheral blood B cells across breast cancer subtypes, pre-NAC B cells trended higher in patients with HER2 + achieving pCR compared to those with residual disease. This observation is in alignment with studies showing that tumor-derived B cell signatures predict response to NAC in HER2 + breast cancer [42], and that enrichment of tumor-infiltrating B cells correlates with improved survival in TNBC [50, 52].

Our study has several strengths, including (1) the use of peripheral blood samples from a prospective clinical trial, with treatment response information, (2) homogeneous treatment that was guideline-concordant at the time of the study, (3) inclusion of all breast cancer subtypes, and (4) the use of a robust CyTOF panel for single-cell immune phenotyping. Limitations include (1) lack of healthy controls, (2) single PBMC timepoint for evaluation, precluding immune phenotype monitoring throughout NAC, (3) inability to further phenotype the myeloid compartment, (4) the use of cryopreserved samples, which may lead to non-proportional loss of cell types more susceptible to the freeze/thaw process, (5) evaluation limited to association of immune phenotype with NAC response (without evaluation of long-term outcomes), and (6) limited sample size impacting the ability to examine separately luminal A from luminal B or ER + HER2 + from ER-HER2 + breast cancer, or to carry out multivariate analyses. Additionally, patients with TNBC in this study were treated prior to the introduction of neoadjuvant immunotherapy, which has since become standard [53]. Further studies longitudinally examining the peripheral blood immune phenotype and the functional state of immune cell populations at various time points throughout NAC and potential associations with long-term clinical outcomes may provide further insights into their potential as a minimally invasive biomarker. A prospective evaluation using freshly stained PBMC samples from patients undergoing modern NAC regimens for breast cancer, and including healthy controls is ongoing in our center (NCT04897009).

### Supplementary Information

**Additional file 1.**

## Data Availability

Data are available upon reasonable request to the corresponding author.
