# Supplementary material for: Pre-treatment peripheral blood immunophenotyping and response to neoadjuvant chemotherapy in operable breast cancer
Source: Breast Cancer Res. 2024 Jun 10;26:97. doi: 10.1186/s13058-024-01848-z (PMC11165781; doi:10.1186/s13058-024-01848-z)
Supplement: Supplementary file 1 — Additional file 1. [file 13058_2024_1848_MOESM1_ESM.docx]

**Supplementary material for “Pre-treatment peripheral blood immunophenotyping and response to neoadjuvant chemotherapy in operable breast cancer”**

**Fig S1:** Relative pre-treatment abundance of B and T immune cell subsets in all patients (N=122)


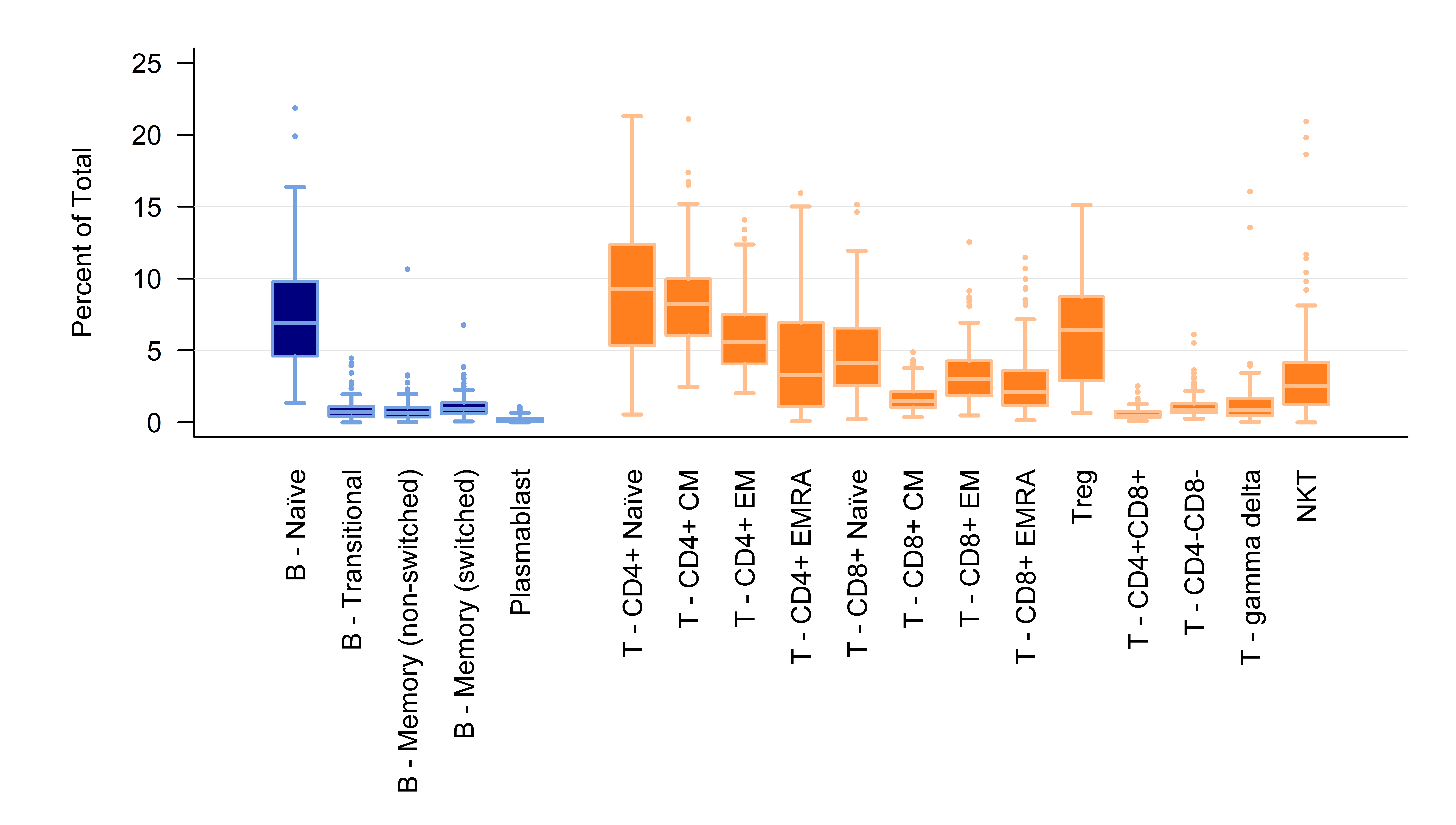


**Fig S2:** Relative pre-treatment abundance of B and T immune cell subsets in patients with TNBC (n=39)

**
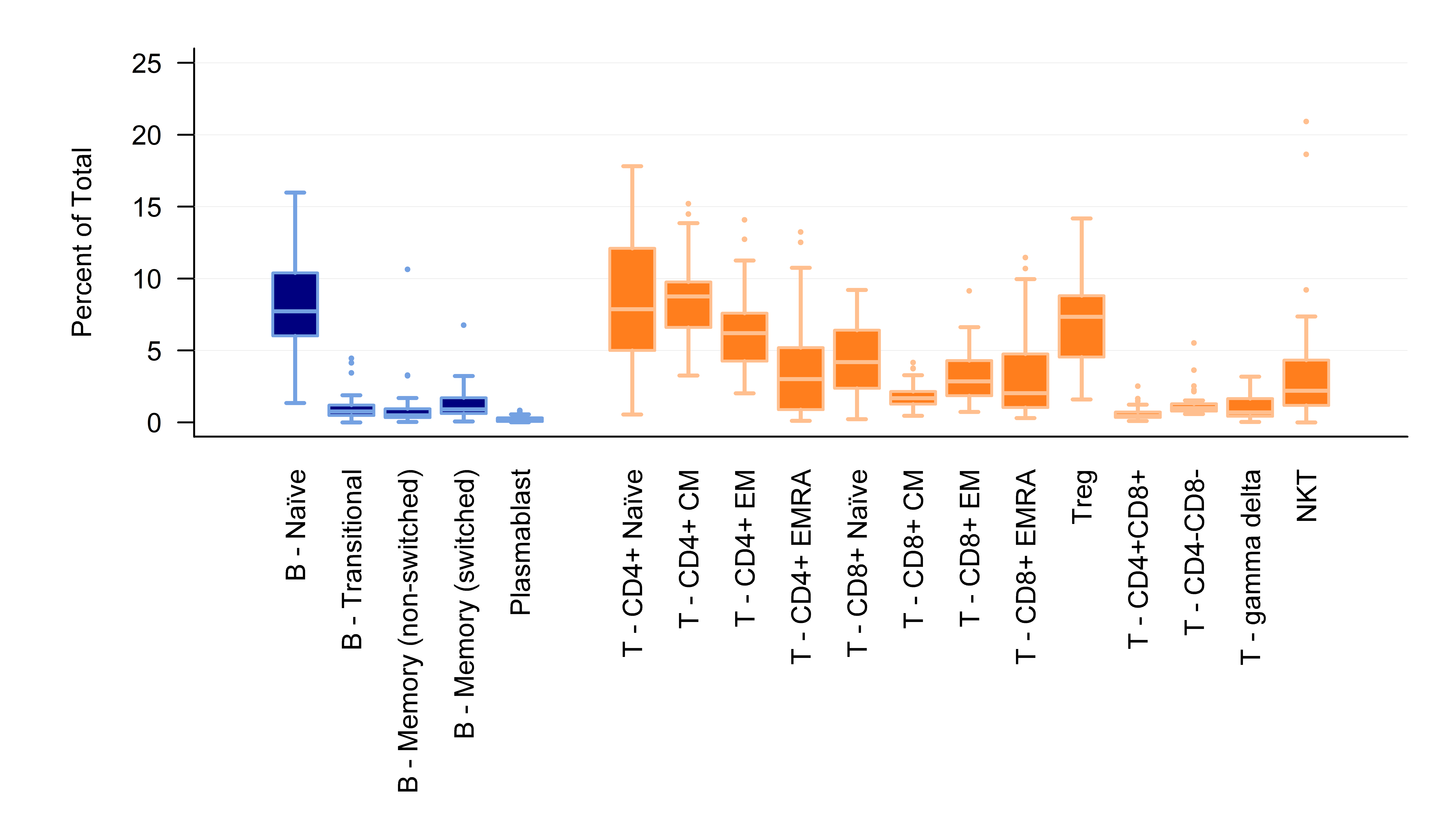
**

**Fig S3:** Relative pre-treatment abundance of B and T immune cell subsets in patients with HER2+ breast cancer (n=36)

**
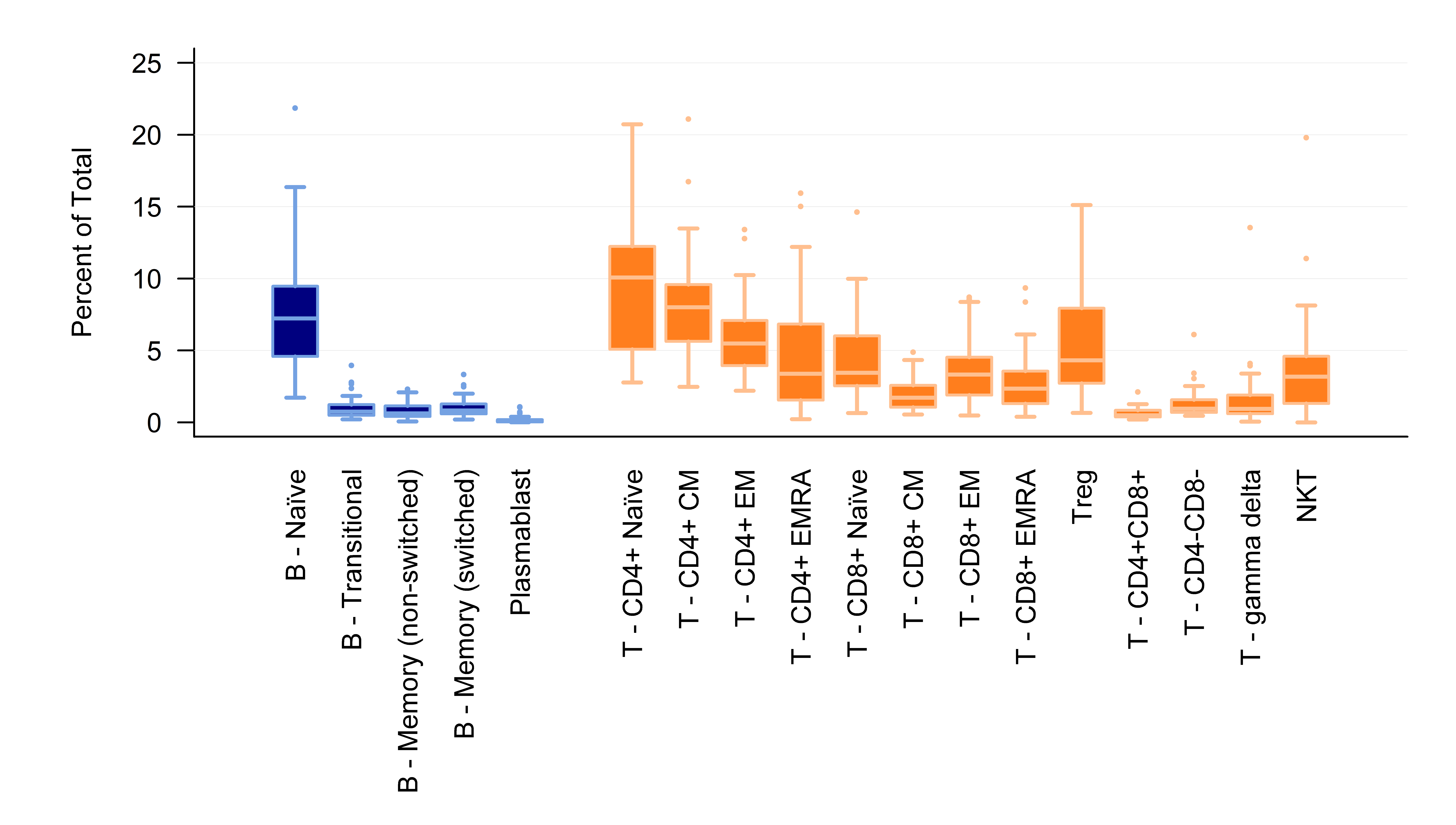
**

**Fig S4:** Relative pre-treatment abundance of B and T immune cell subsets in patients with luminal breast cancer (n=47)

**
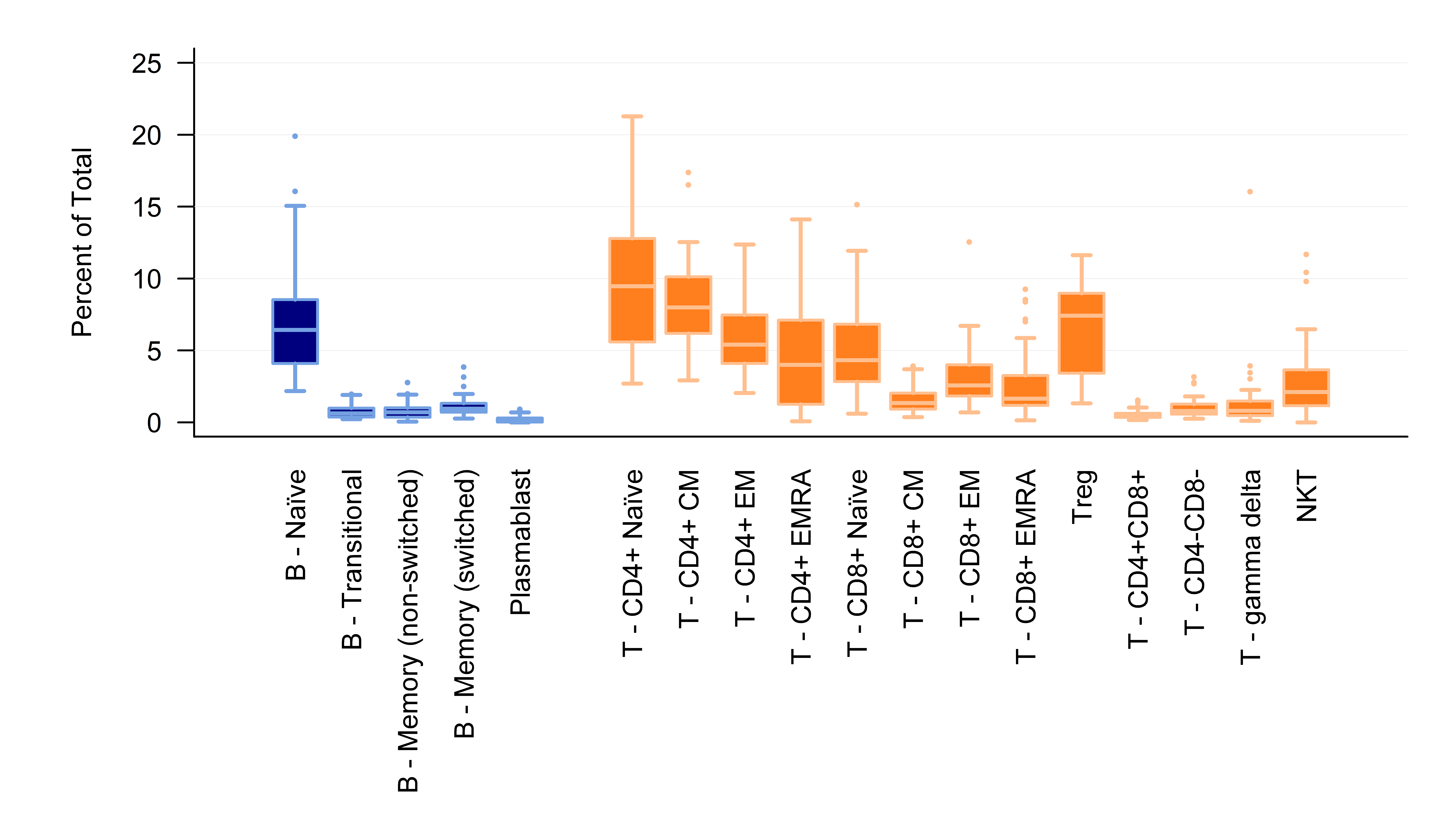
**

**Fig S5:** Relative pre-treatment abundance of B and T immune cell subsets in TNBC according to response to NAC


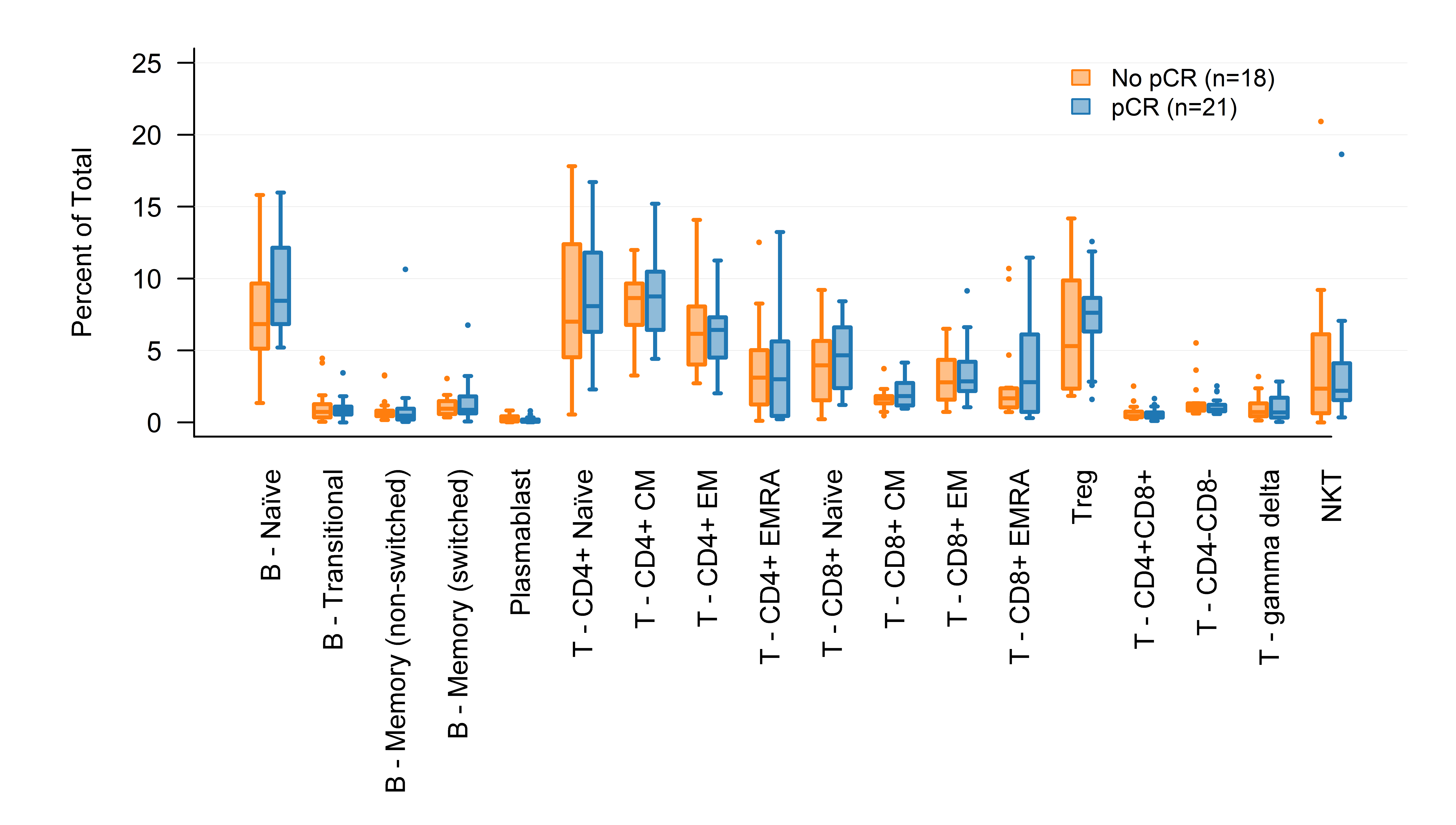


**Fig S6:** Relative pre-treatment abundance of B and T immune cell subsets in Luminal breast cancer according to response to NAC


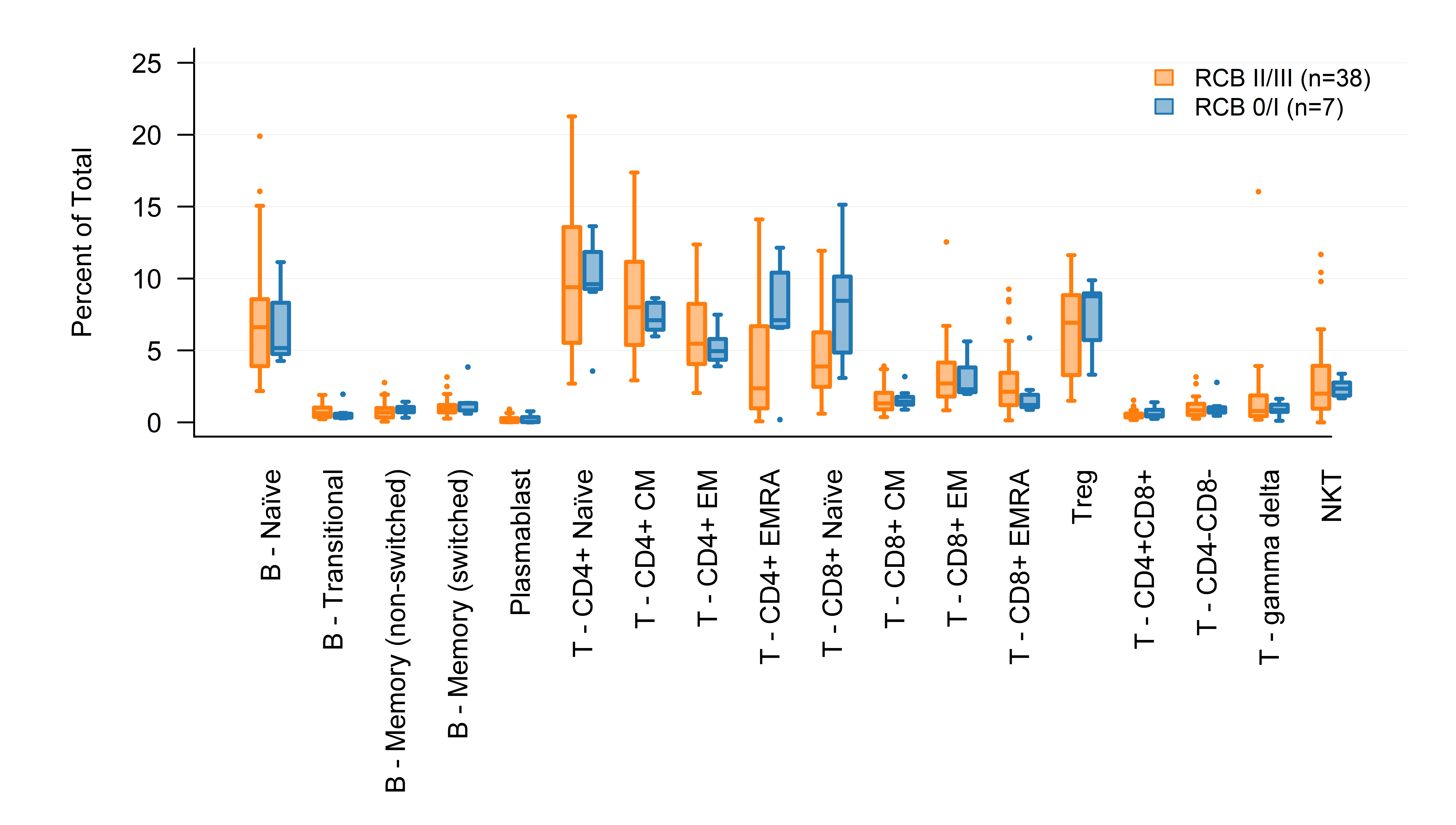


**Fig S7:** Relative pre-treatment abundance of B and T immune cell subsets in HER2+ breast cancer according to response to NAC


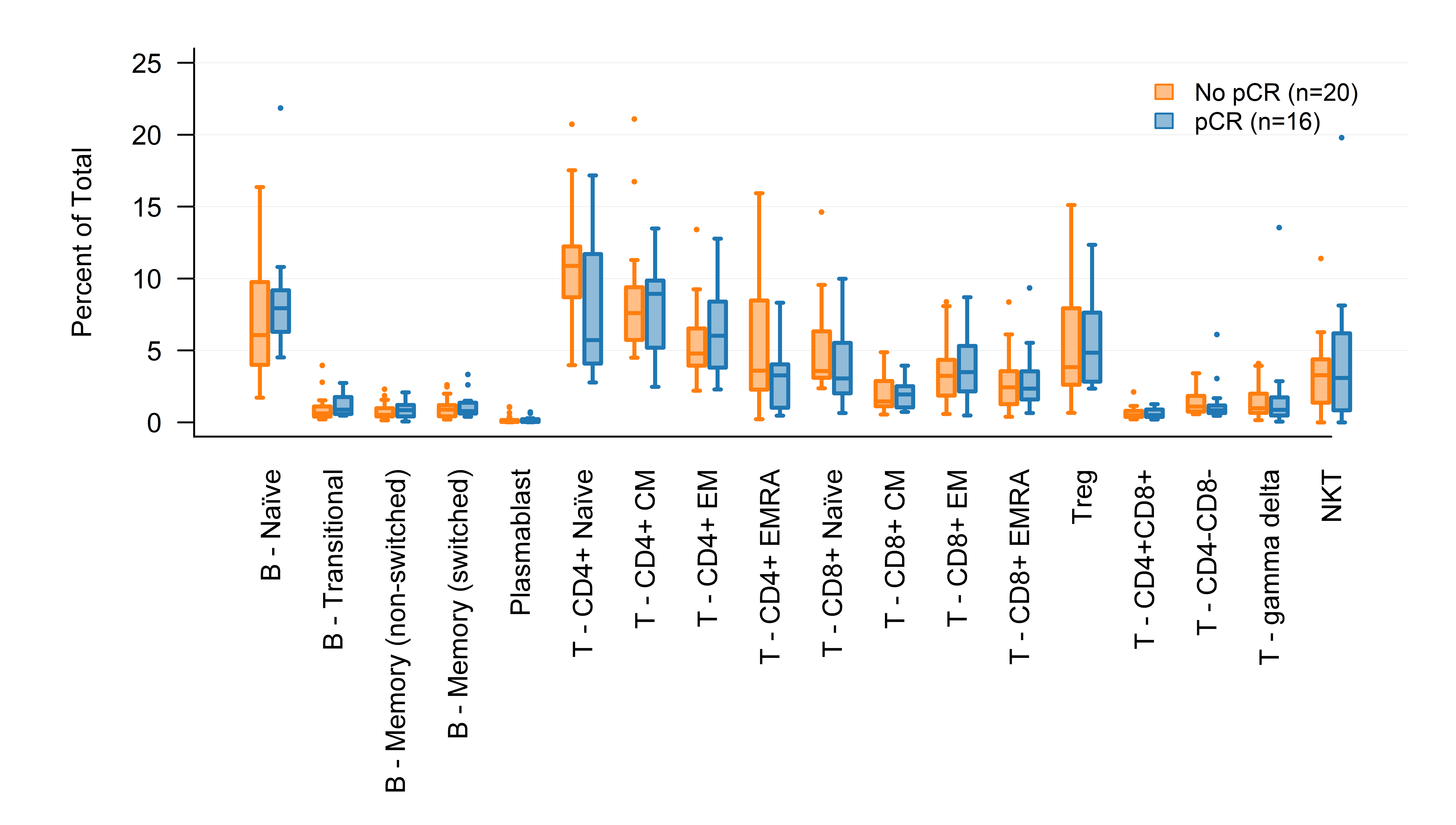


**Fig S8:** Correlation of CD8+ Naïve T cells versus age. (A) Luminal Breast Cancer, (B) HER2+ Breast Cancer, (C) TNBC

**
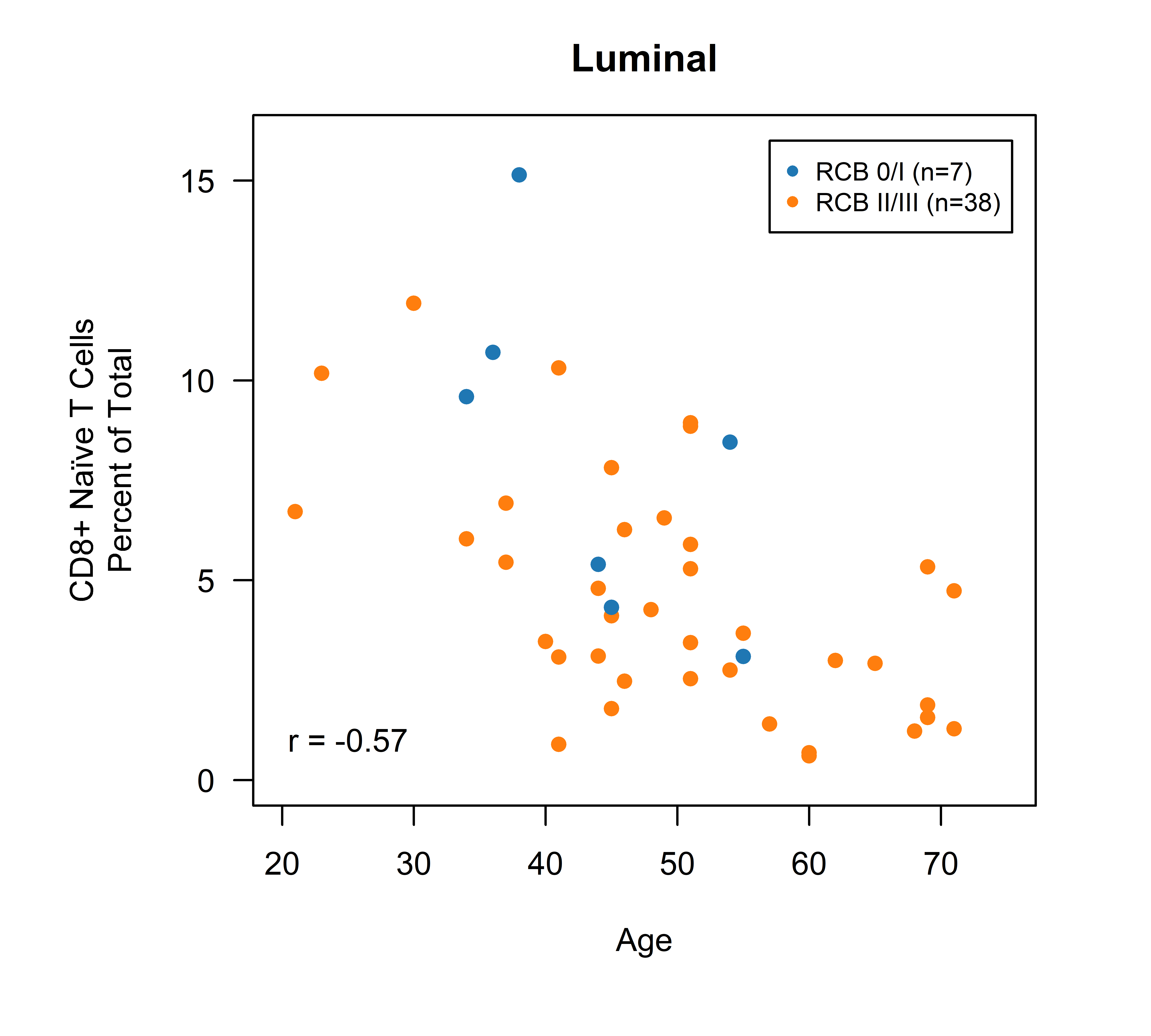

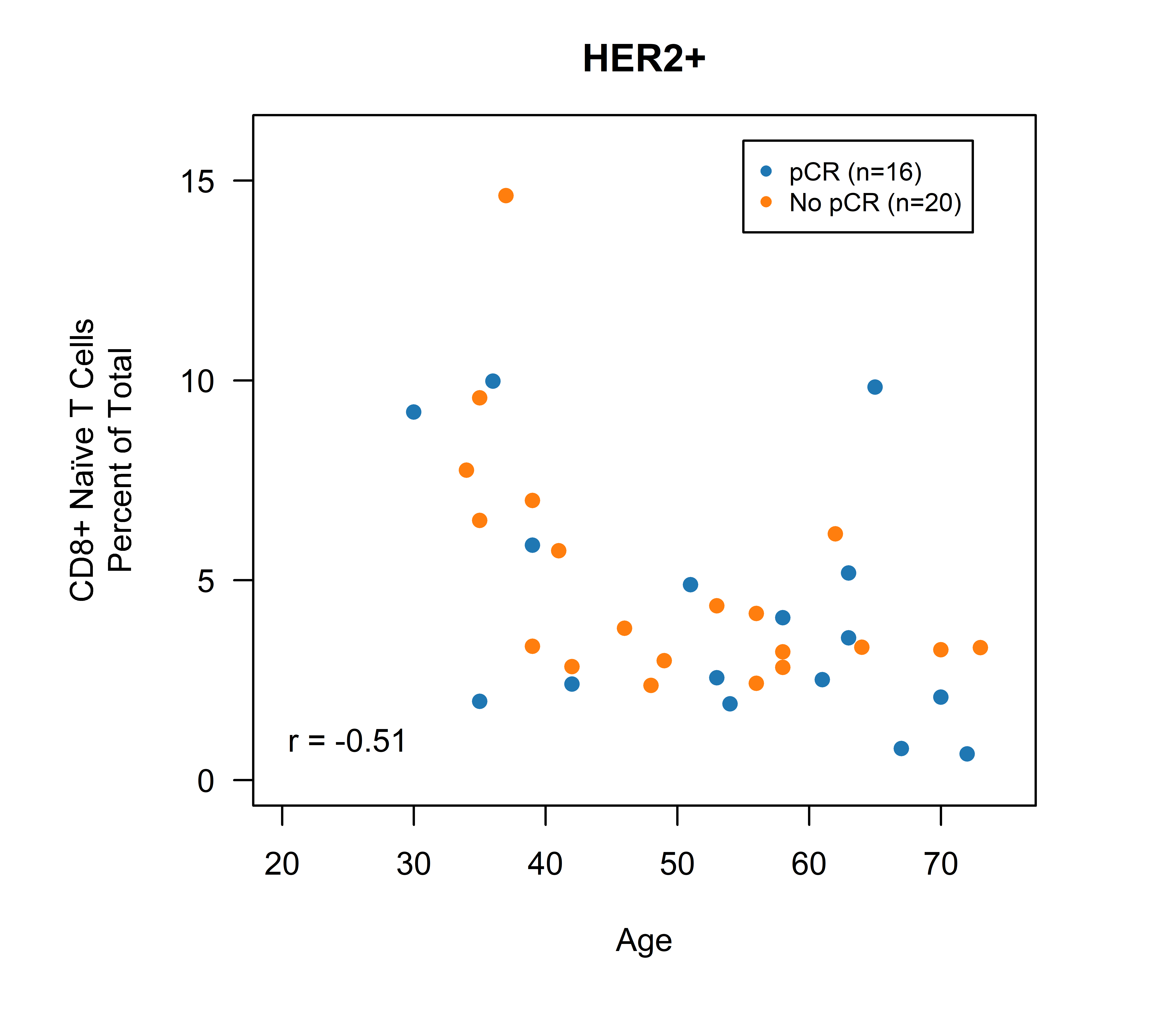
**

**
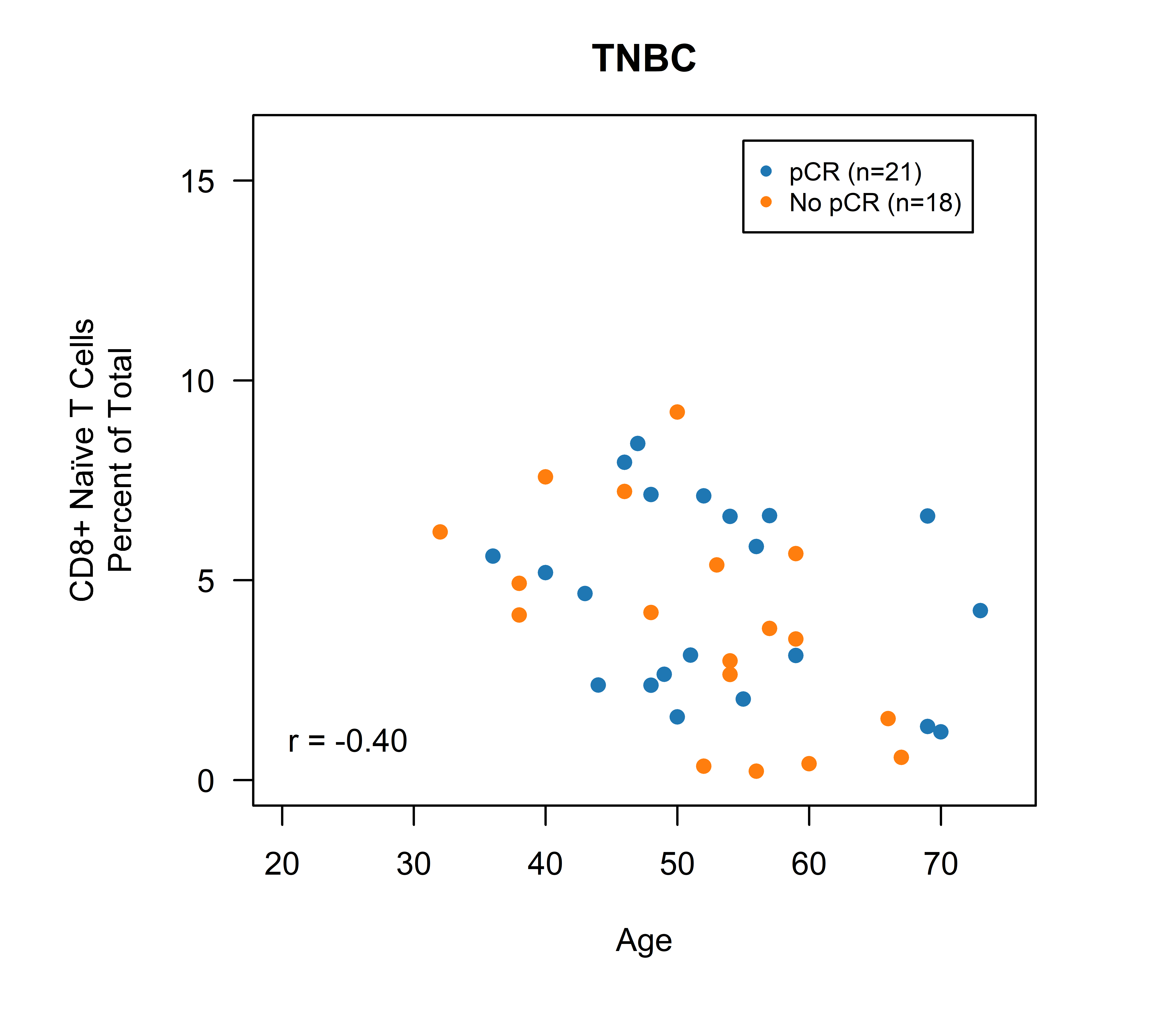
**

**Fig S9:** Correlation of TILs with pCR in TNBC (n=24)

**
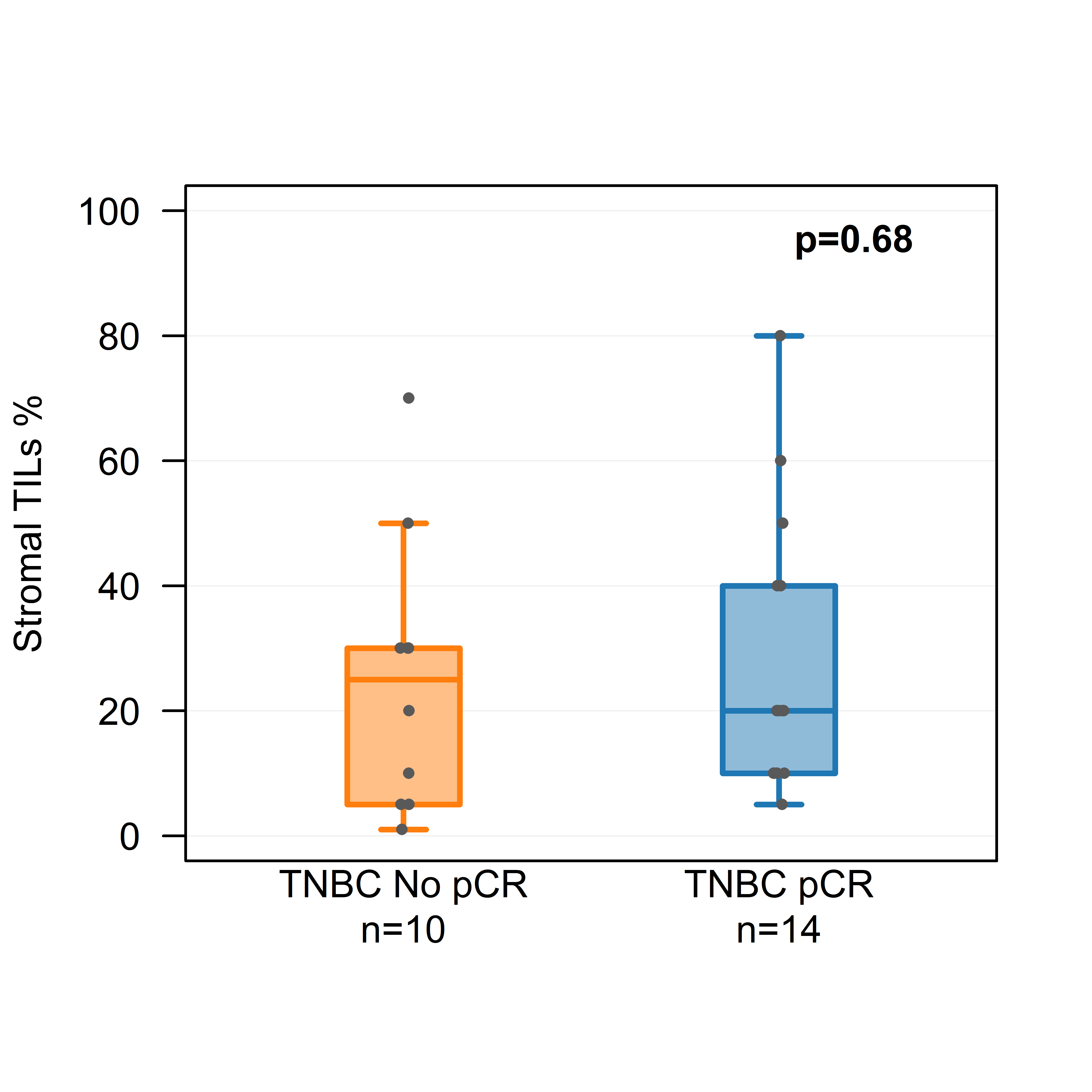
**

**
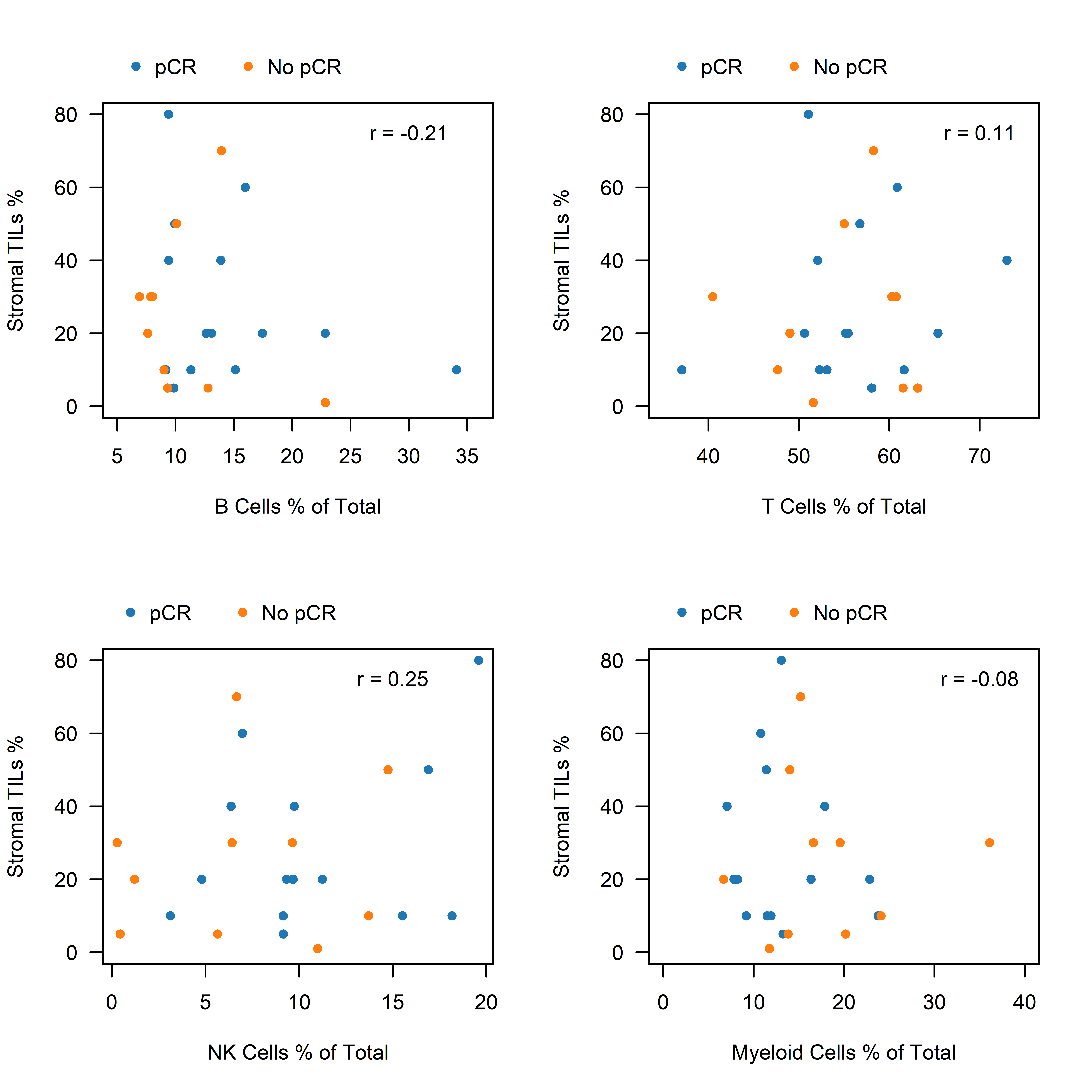
Fig S10:** Correlation of stromal TILs with the abundance of major peripheral blood immune cell subtypes in TNBC (n=24 [pcR: 14, no pCR: 10])

**Fig S11:** Correlation of stromal TILs with the abundance of peripheral blood T cell subtypes in TNBC (n=24 [pcR: 14, no pCR: 10])

**
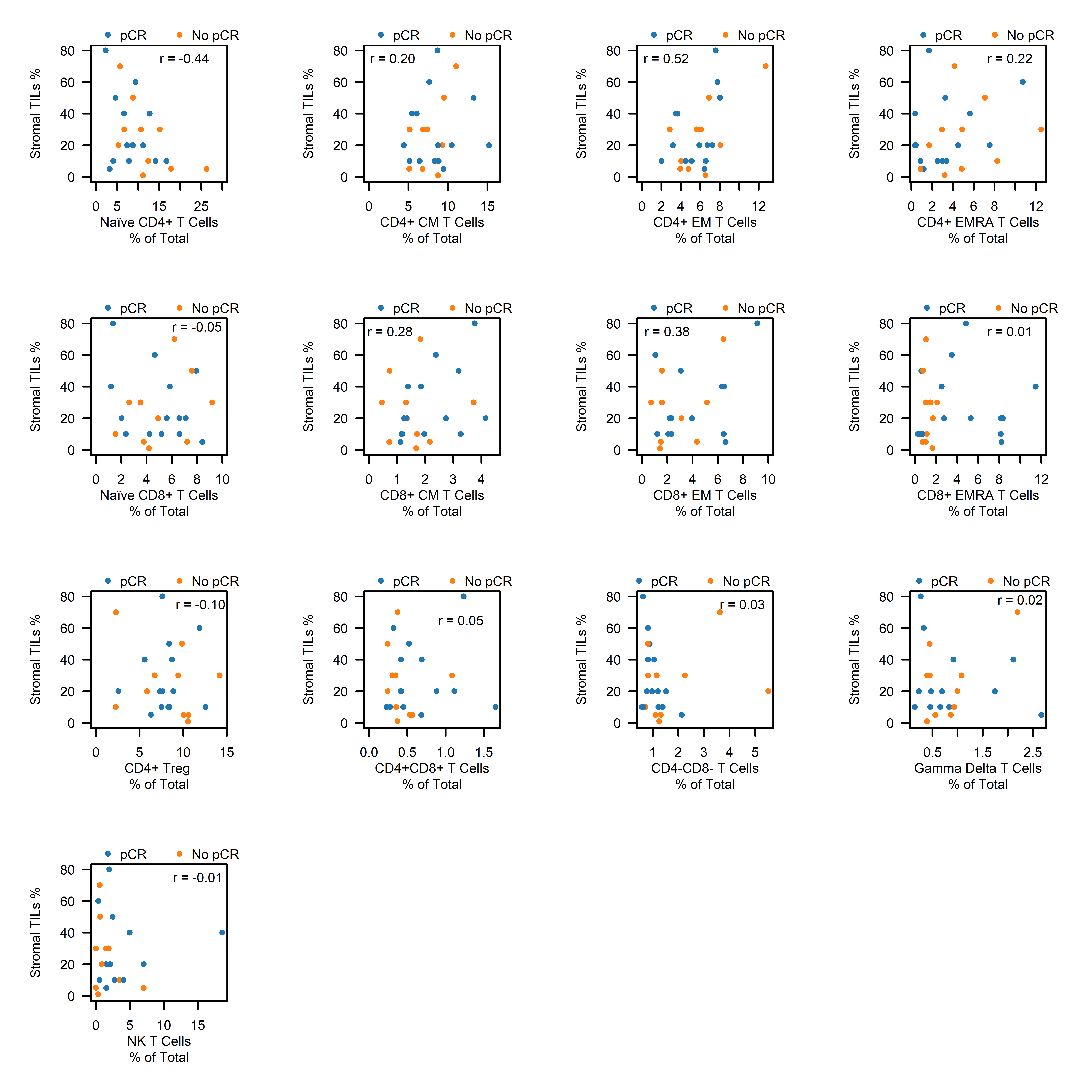
**

**Fig S12:** Correlation of stromal TILs with the abundance of peripheral blood B cell subtypes in TNBC (n=24 [pcR: 14, no pCR: 10])

**

**

**Table S1:** Spearman rank correlations between age and each immune cell subset by tumor subtype

|  | **Luminal** | **HER2+** | **TNBC** |
| --- | --- | --- | --- |
| pcttotal_cd8posNaive | -0.5740 | -0.5060 | -0.3956 |
| pcttotal_cd4negcd8negTcell | -0.4590 | -0.4479 | -0.3867 |
| pcttotal_gdTcell | -0.3846 | -0.3446 | -0.1489 |
| pcttotal_TcellUnassigned | -0.3409 | -0.2755 | -0.0616 |
| pcttotal_cd4posNaive | -0.2561 | -0.1557 | 0.1686 |
| pcttotal_CMnegUnassigned | -0.2472 | 0.2006 | -0.1652 |
| **pcttotal_Unassigned** | -0.2429 | 0.1999 | -0.1697 |
| pcttotal_cd8posEM | -0.2092 | -0.2199 | 0.2089 |
| **pcttotal_Tcell** | -0.1826 | -0.3070 | 0.1915 |
| pcttotal_CD27posBcellUnassigned | -0.1443 | 0.0948 | -0.1973 |
| pcttotal_BcellTransitional | -0.1356 | 0.0574 | -0.0592 |
| pcttotal_GranulocyteBasophil | -0.1014 | 0.0768 | 0.0823 |
| pcttotal_cd8posEMRA | -0.0951 | 0.1460 | 0.4110 |
| pcttotal_cd4posEMRA | -0.0932 | -0.1727 | -0.0141 |
| pcttotal_DendriticCellPlasmacytoid | -0.0796 | -0.3596 | 0.1217 |
| pcttotal_BcellPlasmablast | -0.0365 | -0.0519 | -0.0626 |
| pcttotal_BcellNaive | -0.0122 | -0.0660 | -0.2834 |
| **pcttotal_Other** | 0.0153 | 0.0695 | 0.0569 |
| **pcttotal_Bcell** | 0.0153 | -0.0442 | -0.3285 |
| pcttotal_BcellNonSwitchedMem | 0.0372 | -0.0945 | -0.1761 |
| **pcttotal_MyeloidCell** | 0.0385 | -0.1315 | 0.2294 |
| pcttotal_MyeloidUnassigned | 0.0398 | -0.1098 | 0.2293 |
| pcttotal_cd8posCM | 0.0500 | -0.1301 | 0.0141 |
| pcttotal_RootUnassigned | 0.0512 | -0.1377 | -0.1200 |
| pcttotal_cd4poscd8posTcell | 0.0617 | 0.1667 | 0.4268 |
| pcttotal_CMnegHLADRpos | 0.1214 | 0.0184 | 0.0349 |
| pcttotal_BcellSwitchedMem | 0.1317 | -0.2242 | -0.2185 |
| pcttotal_BcellUnassigned | 0.1472 | 0.2077 | -0.2304 |
| pcttotal_NKTcell | 0.1579 | 0.1118 | 0.2599 |
| pcttotal_NKCellCD56posCD16neg | 0.2266 | 0.4082 | -0.0192 |
| pcttotal_cd4posEM | 0.2757 | -0.2317 | -0.1797 |
| pcttotal_NKCellCD56posCD16pos | 0.2801 | 0.3856 | -0.0186 |
| pcttotal_cd4posTreg | 0.2809 | 0.2466 | -0.0087 |
| **pcttotal_NKCell** | 0.3186 | 0.4623 | -0.0236 |
| pcttotal_cd4posCM | 0.3325 | 0.1681 | -0.1251 |

**Table S2:** Spearman rank correlations between stromal TIL levels and each immune cell subset in TNBC (n=24)

| pcttotal_cd4posEM | 0.52 |
| --- | --- |
| pcttotal_BcellTransitional | 0.45 |
| pcttotal_cd8posEM | 0.38 |
| pcttotal_cd8posCM | 0.28 |
| pcttotal_NKCellCD56posCD16neg | 0.25 |
| pcttotal_NKCell | 0.25 |
| pcttotal_cd4posEMRA | 0.22 |
| pcttotal_cd4posCM | 0.20 |
| pcttotal_NKCellCD56posCD16pos | 0.19 |
| pcttotal_Tcell | 0.11 |
| pcttotal_GranulocyteBasophil | 0.11 |
| pcttotal_Other | 0.06 |
| pcttotal_cd4poscd8posTcell | 0.05 |
| pcttotal_cd4negcd8negTcell | 0.03 |
| pcttotal_gdTcell | 0.02 |
| pcttotal_cd8posEMRA | 0.01 |
| pcttotal_NKTcell | -0.01 |
| pcttotal_CMnegHLADRpos | -0.03 |
| pcttotal_CMnegUnassigned | -0.05 |
| pcttotal_cd8posNaive | -0.05 |
| pcttotal_Unassigned | -0.06 |
| pcttotal_MyeloidUnassigned | -0.08 |
| pcttotal_MyeloidCell | -0.08 |
| pcttotal_cd4posTreg | -0.10 |
| pcttotal_BcellNaive | -0.14 |
| pcttotal_BcellUnassigned | -0.15 |
| pcttotal_RootUnassigned | -0.17 |
| pcttotal_BcellNonSwitchedMem | -0.18 |
| pcttotal_Bcell | -0.21 |
| pcttotal_DendriticCellPlasmacyto | -0.22 |
| pcttotal_TcellUnassigned | -0.23 |
| pcttotal_BcellSwitchedMem | -0.25 |
| pcttotal_BcellPlasmablast | -0.41 |
| pcttotal_cd4posNaive | -0.44 |
| pcttotal_CD27posBcellUnassigned | -0.57 |
